# Unvalued Pearls

**Published:** 1909-05-29

**Authors:** 


### Unvalued Pearls.

Major Ronald Ross, in his lecture delivered at the Royal Institution, complained bitterly of the apathy with which attempts to combat malaria are met in certain ague-stricken British Possessions. In proof of the enormous powers of preventing malaria which the later developments of knowledge have placed in our hands he instanced the case of Italy. Here, under the influence of a campaign which includes the distribution of quinine and the provision of mosquito nets, the deaths from malaria have fallen from 21,000 in 1887 to 4,160 in 1907. In contrast with this he referred to a statement, published in 1907, detailing what has been done in 21 of our Colonies. In seven only, Southern Rhodesia, Papua, Mauritius, British Central Africa, Gambia, Ceylon, and Southern Nigeria, is any real interest taken in the business, while in important colonies like Jamaica the authorities responsible for the public health are shewing a most reprehensible lethargy. In Sierra Leone, which Major Ross visited in 1899 in order to institute, with the benefit of local knowledge, an anti-malaria campaign, practically nothing has been done, and so it is in many other parts of the Empire. Major Ross indicts the heads of the sanitary services of the Colonies, and complains that the Colonial Governments are much too lax in the selection of their sanitary officers, passing over men of approved ability and appointing those whose prime credentials are either mediocrity or subservience. The excuse advanced for the prevailing inactivity is want of funds, yet we are told that "many a town could be kept clear of malaria for the amount, say, of the salary of a single European official." If this be true the plea cannot be accepted, for the provision of healthy conditions of life is not the least of the duties incumbent upon a Government. Altogether Major Ross's address does not suggest a satisfactory state of affairs, and it is to be hoped that his strictures will find attention in the appropriate quarter. It is possible that in his anxiety to reach the fruition of knowledge the speaker has been goaded into stronger language than the occasion demands or the weakness of humanity justifies. We sympathise with him. Nothing is more exasperating than the effort to benefit people who refuse to be benefited, for no task is more thankless. Anyone who has been unwillingly forced into responsibility for a friend who has dined too generously will realise how maddening a thing it is to feel bound in honour to urge reasonable behaviour and yet to receive nothing but jibes in return. The natural indignation which attends such a position—and Major Ross occupies an analogous one towards the recalcitrant colonial health departments—makes warmth of language very venial. But if the charges brought by Major Ross are justified, no time should be lost in bringing all available pressure to bear upon those who in this matter are shirking their elementary obligations.

